# Longtime Vision Function Prediction in Childhood Cataract Patients Based on Optical Coherence Tomography Images

**DOI:** 10.3389/fbioe.2021.646479

**Published:** 2021-03-05

**Authors:** Yifan Xiang, Jingjing Chen, Fabao Xu, Zhuoling Lin, Jun Xiao, Zhenzhe Lin, Haotian Lin

**Affiliations:** ^1^State Key Laboratory of Ophthalmology, Zhongshan Ophthalmic Center, Sun Yat-sen University, Guangzhou, China; ^2^Center of Precision Medicine, Sun Yat-sen University, Guangzhou, China

**Keywords:** machine learning, optical coherence tomography, childhood cataract, visual prediction, intelligent system

## Abstract

The results of visual prediction reflect the tendency and speed of visual development during a future period, based on which ophthalmologists and guardians can know the potential visual prognosis in advance, decide on an intervention plan, and contribute to visual development. In our study, we developed an intelligent system based on the features of optical coherence tomography images for long-term prediction of best corrected visual acuity (BCVA) 3 and 5 years in advance. Two hundred eyes of 132 patients were included. Six machine learning algorithms were applied. In the BCVA predictions, small errors within two lines of the visual chart were achieved. The mean absolute errors (MAEs) between the prediction results and ground truth were 0.1482–0.2117 logMAR for 3-year predictions and 0.1198–0.1845 logMAR for 5-year predictions; the root mean square errors (RMSEs) were 0.1916–0.2942 logMAR for 3-year predictions and 0.1692–0.2537 logMAR for 5-year predictions. This is the first study to predict post-therapeutic BCVAs in young children. This work establishes a reliable method to predict prognosis 5 years in advance. The application of our research contributes to the design of visual intervention plans and visual prognosis.

## Introduction

Normal visual development and visual acuity (VA) are important for young children and are the basis of infantile brain development (Stjerna et al., 2015; Danka Mohammed and Khalil, 2020) and ability development (Wu et al., 2019; Havstam Johansson et al., 2020). Consequently, the results of visual prediction are meaningful by reflecting the tendency and speed of visual development during a future period. Exact VA prediction is beneficial for young children, especially children with ophthalmopathy, based on which ophthalmologists and guardians can determine the potential visual prognosis in advance, decide on an intervention plan, and contribute to visual development. However, the nature of ocular growth and myopia drift in young children may disrupt exact visual prediction and affect result accuracy. To date, no children-applicable technology for VA prediction has been reported. Existing research (Rohm et al., 2018) has focused on short-term visual prediction for adults within a year.

Fundus imaging, especially optical coherence tomography (OCT), is recognized as a key factor for visual prediction (Guo et al., 2017; Esaka et al., 2019; Park et al., 2020). OCT images have been applied to predict prognostic visual function in age-related macular degeneration (AMD) (Rohm et al., 2018) and have achieved excellent performance in visual prediction. In our study, based on clinical data, features of OCT images, and follow-up results of children with childhood cataract (CC), we developed a machine learning system for long-term visual prediction of best corrected visual acuity (BCVA) 3 and 5 years in advance. This system can help ophthalmologists and guardians monitor patients’ visual development (Long et al., 2017) and adopt necessary visual intervention (Sniatecki et al., 2015; Wang and Xiao, 2015) in time, thereby contributing to the visual prognosis of young children.

## Materials and Methods

A prospective study was conducted at the Zhongshan Ophthalmic Center (ZOC), Guangdong, China, from June 2011 to February 2019. The data were collected from a national project for CC treatment and research: the CC Program of the Chinese Ministry of Health (CCPMOH). This study followed the tenets of the Declaration of Helsinki and was approved by the Institutional Review Board of the ZOC at Sun Yat-sen University (IRB-ZOC-SYSU).

### Clinical and Imaging Data

Two hundred eyes of 132 patients from the ZOC were included in the study. The inclusion criteria were as follows: (1) diagnosed with CC at the ZOC, (2) had surgical treatment, (3) had a horizontal OCT B scan with a scan quality index of good, (4) had a clear axis after treatment, and (5) had follow-up BCVA exams at 3 or 5 years after the OCT B scan. The exclusion criteria were as follows: (1) diagnosed with another ophthalmic disease or (2) diagnosed with a neurological or mental disease. The collected clinical data included sex, laterality, surgical age, surgical type, age at OCT B scan, follow-up BCVA results, and other examination and prognostic information.

**FIGURE 1:**
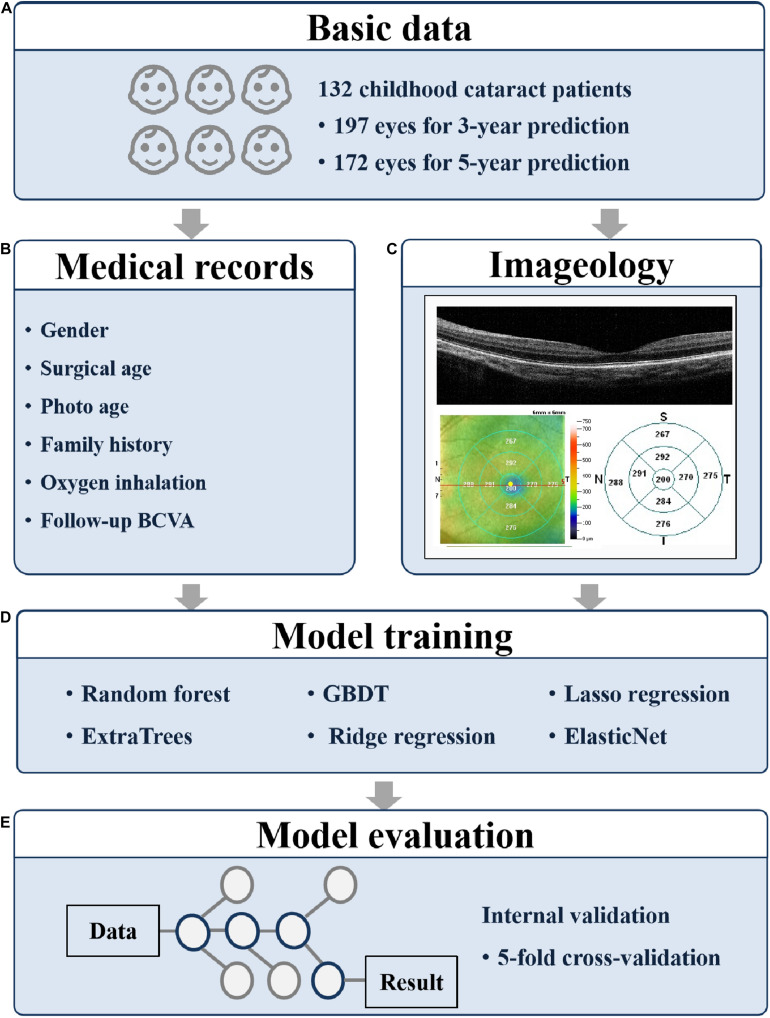
Overall study pipeline. The work flow of developing machine learning models for predicting 3- and 5-year BCVA based on medical records and fundus images. **(A)** The participant numbers; **(B)** the collected medical records; **(C)** the collected fundus images; **(D)** the applied models; and **(E)** the evaluation method. BCVA, best corrected visual acuity; GBDT, gradient boosting decision tree.

Measured features of OCT images were extracted from the Optovue software. The retinal thickness of nine parts, namely the macular area and its eight surrounding regions (inner and outer sides of nasal, temporal, superior, and inferior regions to macula, see Figure 1C), was recorded. Furthermore, the thickness of the retinal nerve fiber layer (RNFL) was labeled manually based on the OCT images. The thickness of the RNFL was divided into four grades. A label of 1 indicated the complete existence of RNFL, 2 indicated the existence of most of RNFL with a thickness more than half of the normal one, 3 indicated the intermittent existence of RNFL with a thickness less than half of the normal one, and 4 indicated the complete absence of RNFL. The label work was finished by three retinal ophthalmologists and confirmed by a retinal professor.

### Model Training and Evaluation

The research procedure is shown in Figure 1. To predict the BCVA of CC patients 3 and 5 years in advance, we tried six machine learning models, namely random forest, ExtraTrees, gradient boosting decision tree (GBDT), ridge regression, lasso regression, and ElasticNet. The age at OCT B scan, the label of RNFL thickness, and the retinal thickness of nine parts obtained from OCT were applied as training features.

For the prediction tasks, we used five-fold cross-validation. The proportion of the training set and the test set were 80 and 20%, respectively. To quantitatively evaluate the prediction performance, we applied two evaluation metrics, mean absolute error (MAE) and root mean square error (RMSE). The MAE is calculated as the average value of the absolute error of the prediction results, which directly reflects the deviation of the predicted values from the actual values. The formula for MAE is as follows:

$$\mathrm{MAE}=\frac{1}{N}\,\sum_{i\,=1}^{N}\left|{\tilde{y}}_{i}-y_{i}\right|$$

The RMSE is the square root of mean square error (MSE). The MSE is calculated as the average value of the square of the error of the prediction results. The RMSE is more interpretable considering its unit consistency with the original variables. The formula for RMSE is as follows:

$$\mathrm{RMSE}=\sqrt{\frac{1}{N}\,\sum_{i\,=1}^{N}{\left({\tilde{y}}_{i}-y_{i}\right)}^{2}}$$

In the above two formulas, *N* is the number of predictions per fold, *y*_*i*_ is the ground truth, and ${\tilde{y}}_{i}$ is the predicted value.

## Results

The training data included 200 eyes of 132 patients (46 females and 86 males), containing 197 eyes of 131 patients for 3-year prediction training and 172 eyes of 114 patients for 5-year prediction training (Table 1). The average surgical age was nearly 47 months, and the average age at OCT B scan was close to 55 months. The average endpoint BCVAs were 0.45 and 0.33 logMAR, respectively, in the two prediction groups.

**TABLE 1:** Characteristics of patients regarding the 3- and 5-year predictions.

**Characteristic**	**3-Year prediction**	**5-Year prediction**
Eyes	197	172
Patients	131	114
Male	85 (64.8%)	76 (66.6%)
Surgical age (months)	46.74 ± 34.17	46.70 ± 34.75
Photo age (months)	55.40 ± 34.12	54.63 ± 34.83
Endpoint BCVA (logMAR)	0.45 ± 0.49	0.33 ± 0.42

*BCVA, best corrected visual acuity.*

For the prediction evaluation, most of the models achieved excellent performance of errors within two lines (0.2 logMAR) of the VA chart in both 3- and 5-year predictions (Table 2). The random forest and GBDT models achieved the best 3-year prediction, and the ExtraTrees and GBDT models achieved the best 5-year prediction. In the 3-year prediction test, the MAEs ranged from 0.1482 to 0.2117, and the RMSEs ranged from 0.1916 to 0.2942. In the 5-year prediction test, the MAEs ranged from 0.1198 to 0.1845, and the RMSEs ranged from 0.1692 to 0.2537. For the same model, the prediction errors of 5-year tasks were always lower than those of 3-year tasks.

**TABLE 2:** Prediction errors in 3- and 5-year BCVA predictions.

**Model**	**3-Year prediction**		**5-Year prediction**	
**Validation set**	**MAE**	**RMSE**	**MAE**	**RMSE**
Random forest	0.2121 ± 0.0153	0.2841 ± 0.0230	0.1685 ± 0.0011	0.2414 ± 0.0019
ExtraTrees	0.2252 ± 0.0009	0.3044 ± 0.0012	0.1558 ± 0.0012	0.2250 ± 0.0015
GBDT	0.2234 ± 0.0024	0.3172 ± 0.0034	0.1807 ± 0.0023	0.2564 ± 0.0026
Ridge regression	0.2253 ± 0.0203	0.2994 ± 0.0322	0.1709 ± 0.0207	0.2566 ± 0.0430
Lasso regression	0.2362 ± 0.0179	0.3073 ± 0.0249	0.1743 ± 0.0139	0.2425 ± 0.0261
ElasticNet	0.2154 ± 0.0176	0.2973 ± 0.0191	0.1719 ± 0.0172	0.2478 ± 0.0308
Test set	MAE	RMSE	MAE	RMSE
Random forest	0.1515 ± 0.0025	0.1916 ± 0.0040	0.1315 ± 0.0031	0.1752 ± 0.0045
ExtraTrees	0.1676 ± 0.0024	0.2108 ± 0.0022	0.1334 ± 0.0019	0.1692 ± 0.0022
GBDT	0.1482 ± 0.0065	0.2024 ± 0.0048	0.1198 ± 0.0040	0.1734 ± 0.0034
Ridge regression	0.2117 ± 0.0401	0.2942 ± 0.0716	0.1475 ± 0.0430	0.2135 ± 0.0664
Lasso regression	0.2046 ± 0.0374	0.2909 ± 0.0768	0.1845 ± 0.0669	0.2537 ± 0.1172
ElasticNet	0.2089 ± 0.0517	0.2937 ± 0.0971	0.1778 ± 0.0345	0.2487 ± 0.0604

*BCVA, best corrected visual acuity; GBDT, gradient boosting decision tree; MAE, mean absolute error; RMSE, root mean square error.*

**FIGURE 2:**
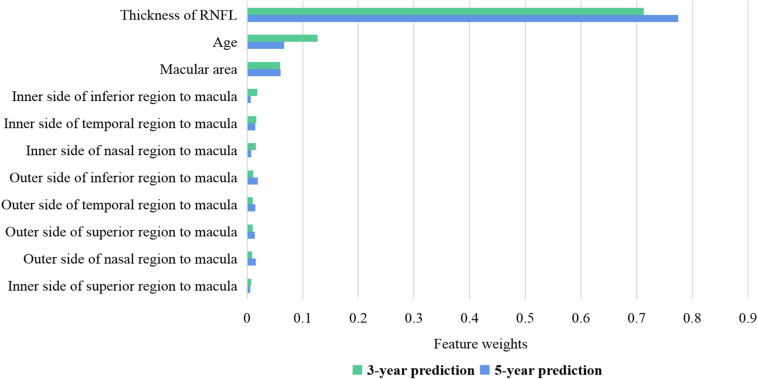
Feature weights of random forest for 3- and 5-year BCVA predictions. The thickness of RNFL has a weight of nearly 0.7–0.8. The age at OCT B scan has a weight of nearly 0.1. The retinal thickness of the macula is the most important of the retinal thickness of the nine regions at and near the macula. BCVA, best corrected visual acuity; RNFL, retinal nerve fiber layer.

Figure 2 shows the weights of features for 3- and 5-year BCVA predictions in the random forest model. The thickness of RNFL plays a key role in BCVA prediction with a weight of nearly 0.8. The age at OCT B scan is the second most important factor with a weight of very nearly 0.1. The retinal thickness of the macula is the most important of the retinal thickness of the nine regions at and near the macula. In the 5-year prediction, the weight of the thickness of RNFL is higher than that of the 3-year prediction. The results of other models are similar to those of the random forest model. The thickness of RNFL remains most important in all the models.

**FIGURE 3:**
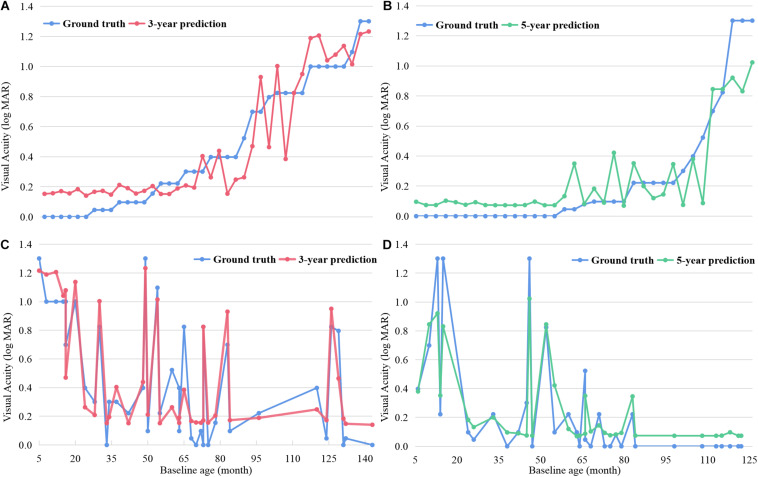
The ground-truth and prediction values of each test example in 3- and 5-year BCVA predictions based on random forest. The examples were ordered based on the ground-truth BCVA in **(A,B)** and ordered based on the age undergoing OCT B scan in **(C,D)**. BCVA, best corrected visual acuity.

Figure 3 shows both the ground-truth and prediction values of each test example in 3- and 5-year predictions, respectively, based on the random forest model. The examples were ordered based on the ground-truth BCVA in Figures 3A,B. When the true BCVAs were lower than 0.2 logMAR, the prediction values were always higher than the true results. As the true BCVAs increased, the prediction values fluctuated around the true results. In Figures 3C,D, the examples were ordered based on the age at OCT B scan, and the prediction values mainly fluctuated around the true results.

## Discussion

This is the first study to predict the post-therapeutic long-term BCVA of CC children based on OCT images through artificial intelligence (AI), and it demonstrated that the long-term visual function of children can be accurately predicted based on imageology using machine learning.

The post-therapeutic visual function of children with ocular diseases is one of the most important factors (Mndeme et al., 2020) focused on by doctors and guardians, as visual prognosis plays a key role in intelligence development (Li et al., 2017), school attendance (Negretti et al., 2015), and quality of life. However, there has been limited research contributing to the visual prediction of children with ophthalmopathy, with most published studies paying attention to the short-term visual prediction of adults within a year (Rohm et al., 2018). Our study addresses both limitations by demonstrating children-applicable prediction and long-term prediction of VA based on imageology. The machine learning models can predict 3- and 5-year BCVAs in advance with a small error within two lines of the visual chart. Based on the results predicted by our model, ophthalmologists and guardians can provide necessary assistance and individually targeted intervention (Sniatecki et al., 2015) to help children obtain better visual outcomes (Pinto et al., 2015) and quality of life, which may be of significant importance to childhood brain development.

Our research achieves precise prediction of long-term BCVA based only on features of OCT images and age, which makes it more accessible and stable than other methods. Most CC patients will take a fundus photo or undergo an OCT B scan to check fundus function after cataract surgery, and our model can be conveniently applied. The reported research (Rohm et al., 2018) applied 165 features to achieve a 6-month prediction, including 41 features from clinical records and 124 features from OCT images. Compared with previous research, our models are simpler and more convenient for general application.

The feature weights shown in Figure 2 specify that the thickness of the RNFL is closely related to long-term visual development after therapy. In the longer prediction, its importance increases. The RNFL lacks the ability to regenerate (Cen et al., 2018; Wu et al., 2020). If the OCT image indicates that the RNFL has atrophied (balducci et al., 2017) at the baseline examination, the visual function would not improve much in the post-therapeutic follow-up. On the other hand, if the OCT image indicates that the RNFL is complete at baseline, CC patients may achieve remarkable visual improvement after surgery with proper intervention. Above all, the thickness of RNFL is a dominant and stable indicator in post-therapeutic BCVA prediction.

Random forest (Breiman, 2001), ExtraTrees (Geurts et al., 2006), and GBDT (Schwenk and Bengio, 1998) all belong to ensemble learning (Kadiyala and Kumar, 2018), in which random forest and ExtraTrees apply the bagging method (Kristína et al., 2006) and GBDT uses the boosting method. Each predicted function was parallel in the bagging model and serial in the boosting model. Bagging always behaves better in preventing overfitting in small sample learning. The models of ridge regression, lasso regression, and ElasticNet (Ogutu et al., 2012) tend to apply the least squares method to predict values, which behaves better in data with multicollinearity and does not exactly fit the weights of each dimension of our data without multicollinearity.

## Limitation

The limitations of our research should be considered. Larger samples of CC patients are necessary to increase the dataset and to improve the prediction stability. Additionally, a longer follow-up period contributes to extending the predicted time span. Besides, data for external validation are warranted to test the prediction model in real-world settings.

## Data Availability Statement

The data is available from the corresponding author upon reasonable request.

## Ethics Statement

The studies involving human participants were reviewed and approved by the institutional review board of the ZOC at Sun Yat-sen University (IRB-ZOC-SYSU). Written informed consent to participate in this study was provided by the participants’ legal guardian/next of kin.

## Author Contributions

YX, JC, and HL conceived and designed the experiments. JC and ZuL collected the data. YX and FX labeled the data. ZeL and JX performed the experiments and analyzed the data. YX wrote the manuscript. JC, FX, and HL revised it. All authors read and approved the final manuscript.

## Conflict of Interest

The authors declare that the research was conducted in the absence of any commercial or financial relationships that could be construed as a potential conflict of interest.
